# Knockdown of SCF^Skp2^ Function Causes Double-Parked Accumulation in the Nucleus and DNA Re-Replication in *Drosophila* Plasmatocytes

**DOI:** 10.1371/journal.pone.0079019

**Published:** 2013-10-24

**Authors:** Paul T. Kroeger, Douglas A. Shoue, Frank M. Mezzacappa, Gary F. Gerlach, Rebecca A. Wingert, Robert A. Schulz

**Affiliations:** Department of Biological Sciences, University of Notre Dame, Notre Dame, Indiana, United States of America; St. Georges University of London, United Kingdom

## Abstract

In *Drosophila*, circulating hemocytes are derived from the cephalic mesoderm during the embryonic wave of hematopoiesis. These cells are contributed to the larva and persist through metamorphosis into the adult. To analyze this population of hemocytes, we considered data from a previously published RNAi screen in the hematopoietic niche, which suggested several members of the SCF complex play a role in lymph gland development. *eater-Gal4*;*UAS-GFP* flies were crossed to *UAS-RNAi* lines to knockdown the function of all known SCF complex members in a plasmatocyte-specific fashion, in order to identify which members are novel regulators of plasmatocytes. This specific SCF complex contains five core members: Lin-19-like, SkpA, Skp2, Roc1a and complex activator Nedd8. The complex was identified by its very distinctive large cell phenotype. Furthermore, these large cells stained for anti-P1, a plasmatocyte-specific antibody. It was also noted that the DNA in these cells appeared to be over-replicated. Gamma-tubulin and DAPI staining suggest the cells are undergoing re-replication as they had multiple centrioles and excessive DNA content. Further experimentation determined enlarged cells were BrdU-positive indicating they have progressed through S-phase. To determine how these cells become enlarged and undergo re-replication, cell cycle proteins were analyzed by immunofluorescence. This analysis identified three proteins that had altered subcellular localization in these enlarged cells: Cyclin E, Geminin and Double-parked. Previous research has shown that Double-parked must be degraded to exit S-phase, otherwise the DNA will undergo re-replication. When Double-parked was titrated from the nucleus by an excess of its inhibitor, geminin, the enlarged cells and aberrant protein localization phenotypes were partially rescued. The data in this report suggests that the SCF^Skp2^ complex is necessary to ubiquitinate Double-parked during plasmatocyte cell division, ensuring proper cell cycle progression and the generation of a normal population of this essential blood cell type.

## Introduction

The study of *Drosophila* hematopoiesis has been an emerging field in recent years, as the fly hematopoietic system has many parallels with that of vertebrates. Among these similarities are the myeloid cell lineage, biphasic nature of hematopoiesis and conserved genes important for proper hematopoietic development [1,2]. These commonalities, along with the advancement of genetic tools, allows for specific genetic manipulation and analysis of individual gene function in hemocyte lineage establishment and blood cell differentiation.

Hematopoiesis in *Drosophila* occurs in two distinct spatiotemporal waves, embryonic and larval. Important to this study is the embryonic wave, which occurs in the head mesoderm and generates mature hemocytes that are present throughout larval development and maintained into the adult stage [3,4]. The larval wave of hematopoiesis occurs in the lymph gland and mature blood cells do not disperse from this tissue until metamorphosis begins [5].

Another similarity between vertebrates and *Drosophila* is that they both have the evolutionarily-conserved myeloid blood cell lineage. Within this lineage in *Drosophila*, there are two types of hemocytes, which are known to arise from a common precursor. These cell types are the plasmatocyte and the crystal cell. Plasmatocytes compose 95% of the cells in the *Drosophila* hemolymph, are involved in phagocytosis of foreign particles, and considered homologous to mammalian macrophages [3,6,7]. Crystal cells compose approximately 5% of the hemolymph cell population and carry out innate immunity via the processes of melanization and wound healing [8-10]. There is also a third lineage of hemocytes, known as lamellocytes, which are rare in wild-type larvae until they are induced to differentiate by parasitic wasp infestation or genetic perturbation [11,12]. Lamellocytes are thought to differentiate from plasmatocytes, as well as lamellocyte precursors present within the sessile hemocyte population [13-15].

Both plasmatocytes and crystal cells divide exactly four times during embryonic stages, until there are approximately 700 plasmatocytes and 36 crystal cells [3]. Although there are approximately 700 hemocytes during late embryonic stages, first instar animals have less than 200 blood cells. These cells will divide several times throughout larval development, until late third instar, when there are 6,000 to 8,000 blood cells in the animal [16]. During the third larval instar, there are between one and two percent of blood cells that stain for anti-phospho-Histone H3 at any given time [5]. This indicates that the cells are in mitosis, which is when Histone H3 is phosphorylated. 

In order to study the importance of individual genes for the production and proliferation of circulating hemocytes, an *eater* blood cell-specific transcriptional enhancer was utilized as a driver for gene function knockdown experiments. Eater is an EGF-rich phagocytic receptor expressed solely in mature plasmatocytes. The receptor is known to be involved in antigen recognition and its expression is regulated by the GATA factor Serpent [15,17]. Generation of a stable *eaterGal4>UAS-GFP* line allowed us to identify, by directed RNAi knockdown, the SCF complex members that function in plasmatocyte development. The SCF complex is a ubiquitin ligase complex that has one of each of the core family proteins: Skp, Cullin, and F-Box, each of which have multiple members, and are important in substrate specificity [18,19]. Identified by a novel enlarged cell phenotype, the specific SCF complex members that function in hematopoiesis consist of Lin19, SkpA, Roc1a, Skp2 and Nedd8. These giant cells are P1-positive, indicating they are of plasmatocyte origin. Nuclei of these cells were also enlarged, suggestive of over-replication of DNA. Gamma-Tubulin staining indicated the giant cells have multiple centrioles and DAPI staining shows greatly increased DNA content. These findings support the hypothesis that disruption of the SCF complex causes continued cycles of DNA replication without a normal progression to cytokinesis—a phenotype that could be explained by a mechanism of re-replication, or the re-firing of replication origins within the S-phase. An alternative explanation is through a process known as endoreplication, in which the cell would undergo cycling without entry into mitosis. We found that these enlarged cells are enriched in BrdU staining, but are phospho-Histone H3-negative, suggesting that they have completed multiple S-phases and may not have undergone mitosis. Further analysis revealed that knockdown of any member of this specific SCF complex alters the subcellular localization of several cell cycle proteins: Cyclin E, Geminin (Gem) and Double-parked (Dup). These data suggest that abrogation of SCF complex components leads to mitotic defects by inhibiting the degradation of Dup. Additionally, the aberrant phenotypes caused by the SCF knockdown could be partially rescued by overexpressing Gem, the inhibitor of Dup. Together, these data implicate that this SCF^Skp2^ complex is necessary for the degradation of Dup, allowing for proper progression of plasmatocytes through the cell cycle.

## Results

### A Plasmatocyte-Specific RNAi Knockdown Strategy Identifies *lin19* as a Novel Regulator of *Drosophila* Circulating Hemocytes

**Figure 1:**
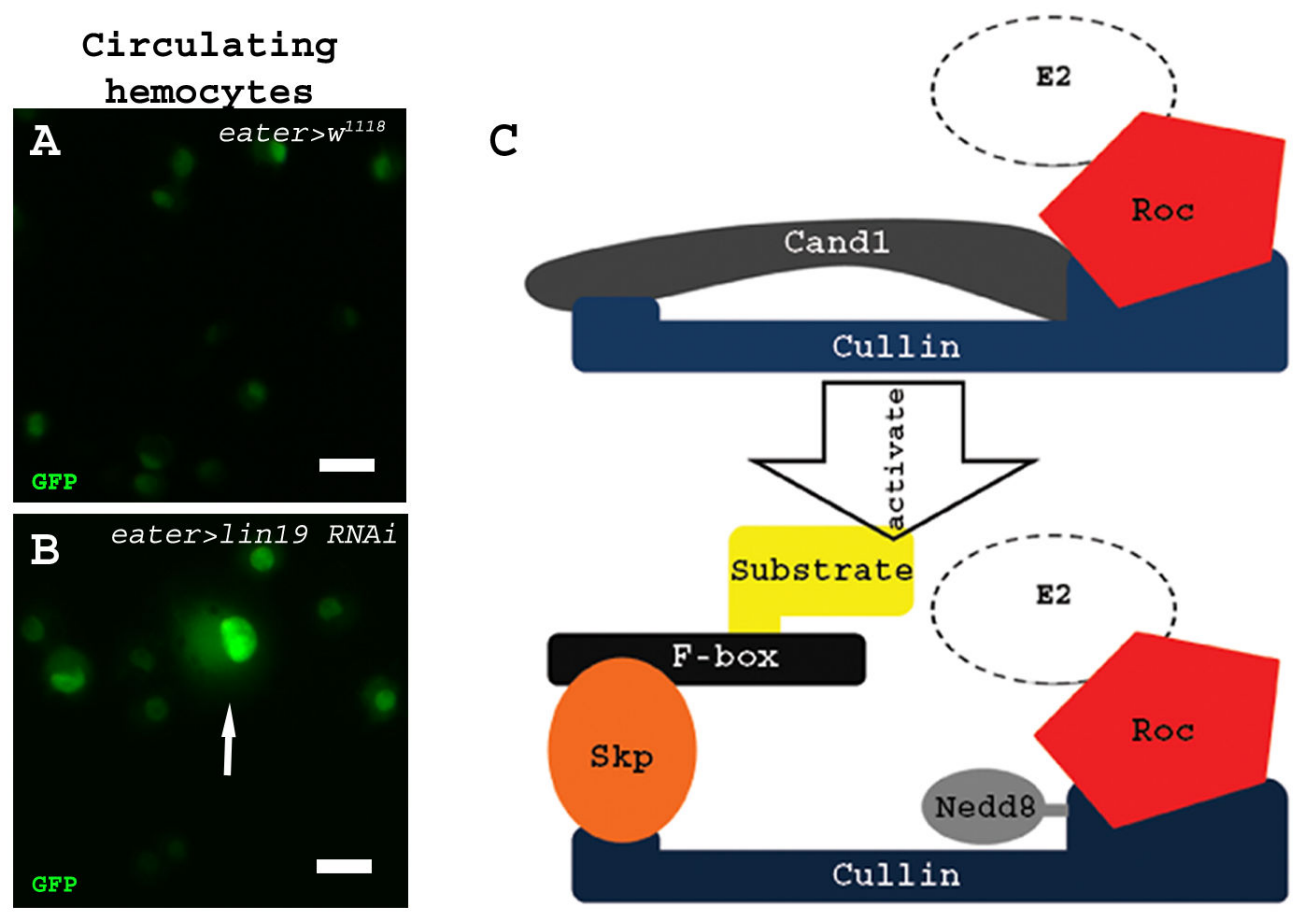
Loss-of-function of *lin19*, a member of the SCF complex, results in an enlarged plasmatocyte phenotype. (A) *eaterGal4>UAS-GFP* is present in control larval circulating hemocytes. (B) *eaterGal4>UAS-GFP>lin19* *RNAi* ^*HM05197*^ hemolymph samples show GFP-positive enlarged cells, indicated by the arrow. (C) Cullin-1 (*Drosophila* homolog is Lin19) belongs to the SCF ubiquitin ligase complex. The SCF complex is inhibited by Cand1 binding to Cullin. To activate the complex, Cand1 is displaced by Nedd8 allowing the Skp/F-box protein pair to bind Cullin giving specificity to the complex to ubiquitinate the target substrate. Scale bars = 20 μm.

 Previously, we isolated an *eater* enhancer necessary to direct GFP reporter expression in mature plasmatocytes [16]. We used this enhancer to generate an *eaterGal4* line of flies for use within the UAS-Gal4 system, to selectively knockdown gene function using a *UAS-RNAi* strategy [15,20]. To visualize plasmatocytes, *UAS-GFP* was crossed into the *eaterGal4* background. Hemolymph samples of RNAi knockdown animals were obtained and compared to *eaterGal4>UAS-GFP*>*w* ^*1118*^ control samples for any aberrations in plasmatocytes, including cell size and cell number (Figure 1A). We had previously shown that *lin19* knockdown caused aberrant lymph gland posterior signaling center (PSC) development [21]. Thus, we investigated whether the knockdown of *lin19* might affect plasmatocytes in circulation. *lin19* was identified as a regulator of cell size in circulating hemocytes, as evidenced by the approximately 5% of cells which are vastly enlarged in hemolymph samples obtained from *eaterGal4>UAS-GFP*>UAS-*lin19 RNAi* ^*HM05197*^(Figure 1B). Lin19 belongs to the SCF ubiquitin ligase complex, which contains members of the Skp, Cullin, F-box and Roc families of proteins. This complex is inactivated by its inhibitor, Cand1, which interacts with the Cullin protein when it is unneddylated, blocking the Skp/F-box protein combination from interacting with Cullin. Nedd8 causes the dissociation of Cand1 by forming a covalent bond with lin19, allowing the complex to become active by binding of the Skp/F-box protein combination, which in turn blocks Cand1 from binding (Figure 1C; [22-24]). Activation of the SCF complex causes ubiquitination of its substrate ultimately leading to proteosomal degradation. 

### SCF Complex Members Which Caused Hematopoietic Aberrations Exhibit a Greatly Enlarged Plasmatocyte Phenotype

**Figure 2:**
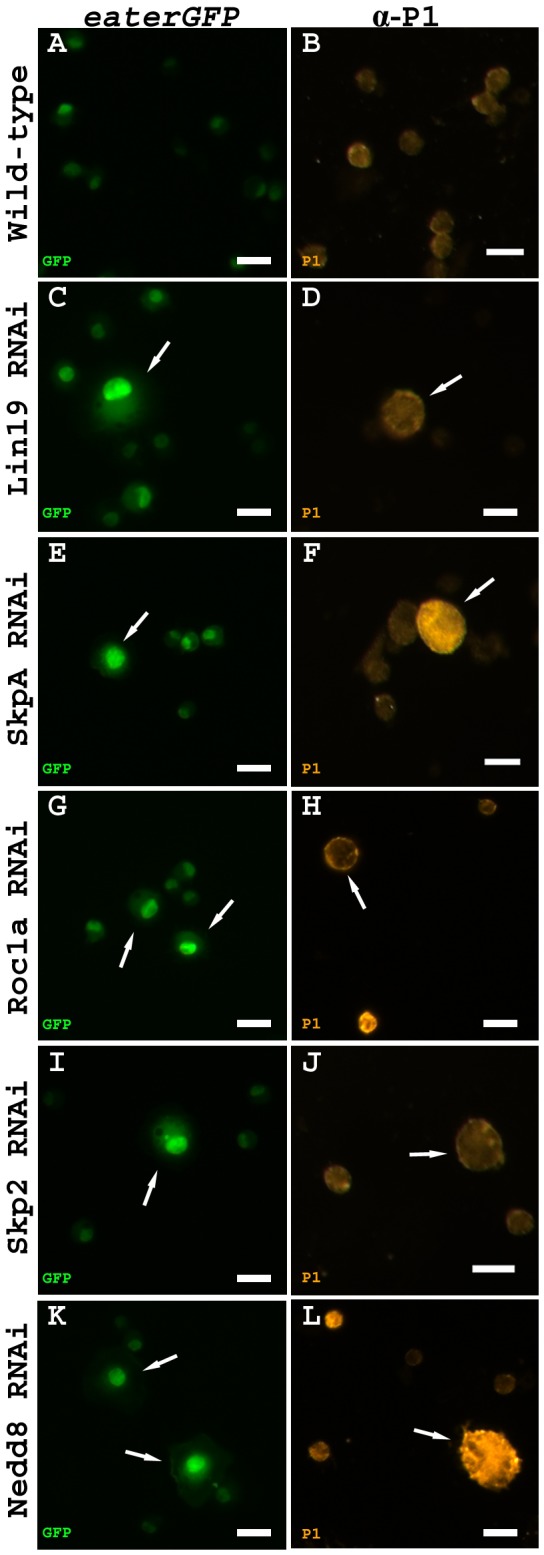
An enlarged plasmatocyte phenotype elicited by RNAi knockdown of members of a specific SCF complex. (A) Control *w* ^1118^ circulating plasmatocytes are marked by an *eaterGal4>UAS-GFP* transgene. (C,E,G,I,K) *eater>GFP>UAS-gene* *RNAi* expressed in circulating hemocytes causes enlarged GFP-positive cells indicated by arrows. (B) *pxn*>*w* ^1118^ control hemocytes stained with anti-P1, a plasmatocyte specific antibody. (D,F,H,J,L) *pxn*>UAS*-gene* *RNAi* hemolymph samples stained with anti-P1 detects enlarged cells labeled with the plasmatocyte-specific antibody indicated by arrows. Scale bars = 20 μm.

 After identification of *lin19* as a regulator of hemocyte size, we next sought to determine the remaining members of this specific SCF complex, which function in hematopoiesis. The majority of known or predicted members of SCF complexes were tested via RNAi functional knockdown or mutant analysis (Table S1, S2). Through these genetic analyses, only one member of each of the core families was determined to cause an enlarged cell phenotype, as observed in *lin19* RNAi knockdown. These findings are consistent with the notion that the SCF complex acting during hematopoiesis consists of SkpA (Skp), Roc1a (Roc), Skp2 (F-box) and Nedd8, as knockdown of these genes, similar to knockdown of *lin19*, also caused approximately 5% of hemocytes to become 2-6 times the size of control cells as shown in *eaterGal4>UAS-GFP>UAS-SCF component RNAi* blood cell samples (Figure 2A,C,E,G,I,K).

 It was hypothesized that the cells becoming enlarged in the RNAi knockdown of this SCF complex were plasmatocytes because we used *eaterGal4* to express the RNAi in a plasmatocyte-specific fashion. However, to determine the hemocyte type that is enlarged in hemolymph samples, we performed anti-P1 plasmatocyte-specific antibody staining using *PeroxidasinGal4* (*PxnGal4*), a similar but stronger driver than *eaterGal4* (Figure 2B,D,F,H,J,L). All enlarged cells that were not lamellocytes were P1-positive suggesting knockdown of each member of this specific SCF complex caused some plasmatocytes to become vastly enlarged. 

### Enlarged Plasmatocytes Possess Multiple Centrioles and Excessive DNA content

**Figure 3:**
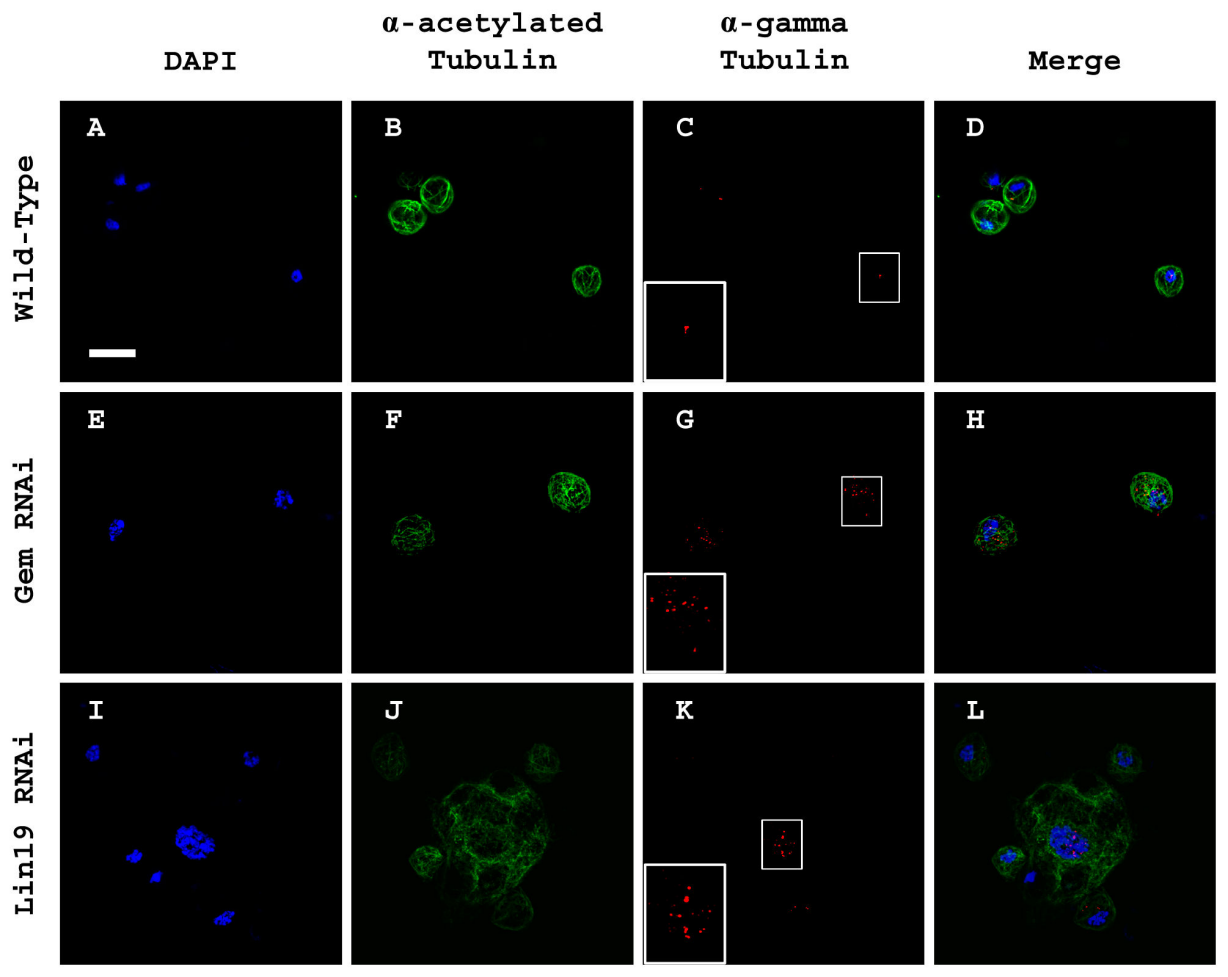
Cells with over-replicated DNA and multiple centrioles are formed after knockdown of SCF complex member *lin19*. (A-D) *pxnGal4>w* ^*1118*^ control hemolymph samples stained with (A) DAPI, (B) α-acetylated-Tubulin, (C) α-gamma-Tubulin, and (D) merge. (E-H) *pxnGal4>UAS-gem* *RNAi* ^*M4*^ positive re-replication control hemolymph samples were stained with (E) DAPI, (F) α-acetylated-Tubulin, (G) α-gamma-Tubulin, and (H) merge. (I-L) *pxnGal4>UAS-lin19* *RNAi* ^*HM05197*^ hemocytes were stained with (I) DAPI, (J) α-acetylated-Tubulin, (K) α-gamma-Tubulin, and (L) merge. Inserts in C, G, and K are a 100% α-gamma-Tubulin magnification. Scale bar = 10 μm.

 During our study of the SCF complex functional knockdown, it was noted that not only were the cells vastly enlarged, but the nuclei of these cells were also sizably larger than wild-type, strongly suggesting DNA over-replication. To investigate this possibility, hemocytes were stained with DAPI and antibodies directed against acetylated-Tubulin and gamma-Tubulin to visualize DNA, microtubules and centrioles, respectively (Figure 3A-D). Wild-type hemocytes showed normal DNA staining (Figure 3A), with one centriole (Figure 3C). It has previously been described that knockdown of *gem* function causes re-replication with overduplication of centrioles (Figure 3E-H; [25]). In *gem* loss-of-function plasmatocytes, it was evident that the DNA had replicated improperly (Figure 3E), and the cells had multiple centrioles (Figure 3G), both indicators of DNA re-replication. Interestingly, functional knockdown of *lin19* caused extensive DNA over-replication (Figure 3I,L), along with a massive excess of centrioles as indicated by gamma-Tubulin staining (Figure 3K). It was also evident that such cells were enlarged, approximately 4 times the size of wild-type, as shown by acetylated-Tubulin staining (Figure 3J). The similarities between the *gem* and *lin19* knockdown conditions suggested that knockdown of the SCF complex caused the cell to undergo substantial re-replication, with excess DNA as well as multiple centrioles present in the nucleus of enlarged plasmatocytes.

### Excessive BrdU Incorporation in Nuclei of Enlarged Plasmatocytes Suggests Increased DNA Replication

**Figure 4:**
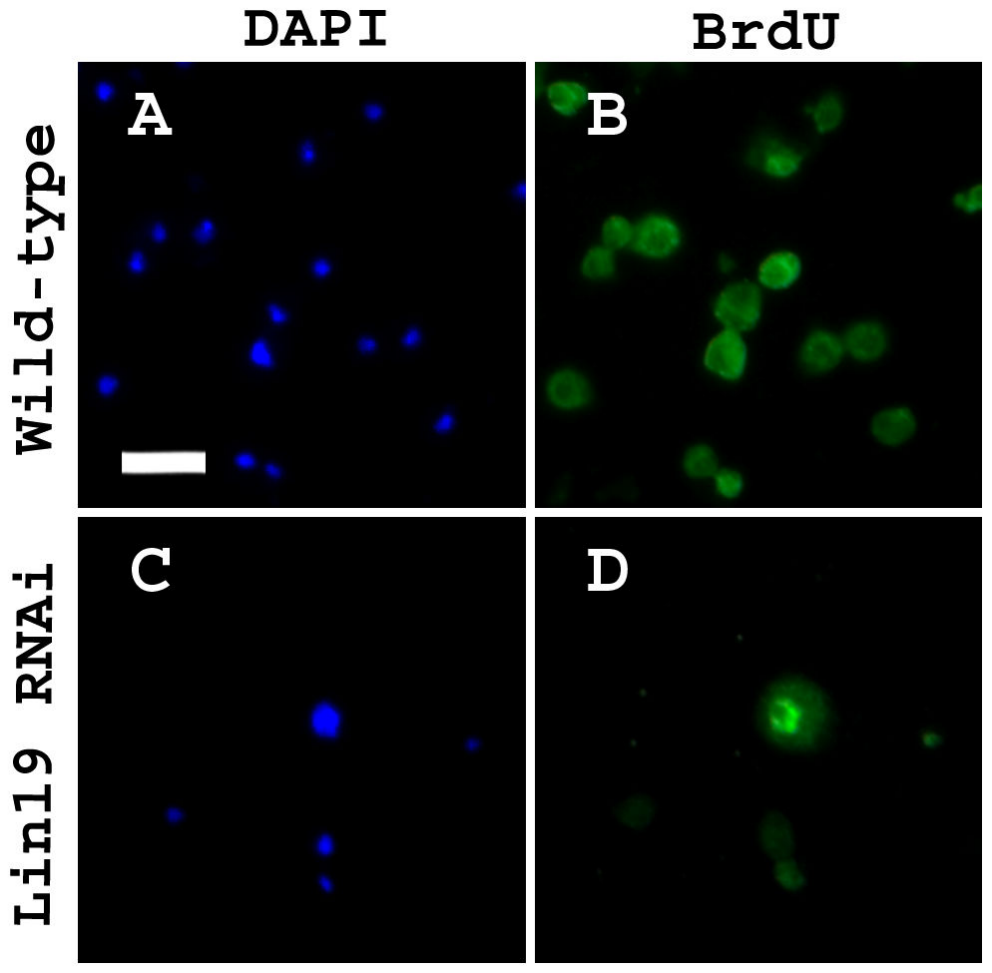
Enlarged cells elicited by functional knockdown of *lin19* exhibit increased nuclear BrdU staining. (A,B) *PxnGal4>w* ^*1118*^ wild-type animals were fed BrdU during larval stages resulting in slight incorporation into the DNA as visualized by anti-BrdU and DAPI staining. (C,D) *pxnGal4>lin19* *RNAi* ^*HM05197*^ were fed BrdU during laval stages resulting in a distinct nuclear BrdU staining in the nucleus when observed with DAPI staining. Scale bar = 20 μm.

 To further examine the possibility of re-replication, BrdU was fed to animals for the duration of larval development until the wandering third instar stage, when larval hemolymph samples were obtained. Increased incorporation of BrdU into DNA suggests an increased number of rounds of DNA synthesis during this developmental time period. Wild-type larvae exhibited a limited amount of BrdU staining in the nucleus, as defined by DAPI staining (Figure 4A,B). Interestingly, in *lin19* knockdown hemocyte samples, the nuclei of enlarged cells were positive for BrdU, indicating that these cells had undergone several rounds of replication (Figure 4C,D). Giant cells did not stain for anti-phospho-Histone H3, suggesting they did not go through mitosis (data not shown). Together, these results are indicative of enlarged cells completing multiple synthesis phases, but not completing a proper mitosis or cytokinesis.

### Knockdown of SCF Complex Members Causes Subcellular Mislocalization of Cell Cycle Proteins Eliciting the Enlarged Plasmatocyte Phenotype

**Figure 5:**
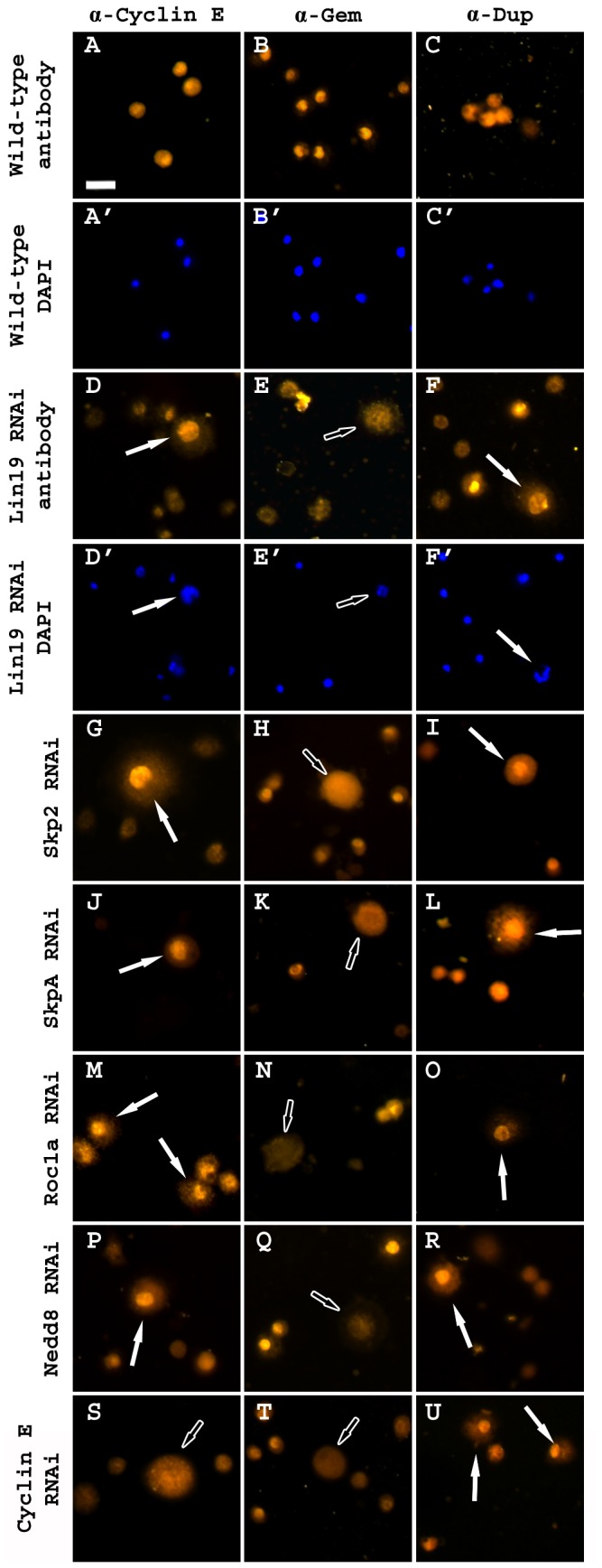
Knockdown of SCF complex members causes changes in subcellular localization of cell cycle regulatory proteins. (A-C) *pxnGal4>w* ^*1118*^ control hemolymph samples stained with (A) α-Cyclin E, (B) α-Gem, and (C) α-Dup and DAPI (A’-C’). (D-F) *pxnGal4>UAS-lin19* ^*HM05197*^ stained with (D) α-Cyclin E, (E) α-Gem, and (F) α-Dup and DAPI (D’-F’). (G-R) *pxnGal4>UAS-SCF* *complex* *member* *RNAi* hemocytes stained with (Column 1) α-Cyclin E, (Column 2) α-Gem, and (Column 3) α-Dup. (S-U) *pxnGal4>UAS-Cyclin* *E* *RNAi* ^*JF02473*^ hemocytes stained with (S) α-Cyclin E, (T) α-Gem, and (U) α-Dup. Nuclear stained enlarged cells are indicated by white filled arrows cytoplasmic stained enlarged cells are indicated by outlined arrows. Scale bar = 20 μm.

 To determine the cause of DNA re-replication and the enlarged plasmatocyte phenotype, several proteins that regulate the cell cycle were assayed by immunohistochemistry of hemolymph samples. Previous research suggested that these phenotypes could be a result of aberrant cell cycle regulation, such as through alterations in Cyclin E and Gem protein localization [25,26]. Wild-type hemolymph staining of anti-Cyclin E showed that Cyclin E was distributed throughout the cytoplasm, when compared to DAPI nuclear stain (Figure 5A,A’). It was also determined that Gem was restricted to the nucleus by the co-localization of DAPI and anti-Gem staining of wild-type hemocytes (Figure 5B,B’). Interestingly, RNAi knockdown of *lin19* caused these two proteins to switch subcellular localization, with Cyclin E increasing its co-localization with DAPI and Gem being exported from the nucleus to the cytoplasm away from the DAPI signal (Figure 5D,D’,E,E’). Furthermore, RNAi knockdown of the remainder of the SCF complex caused protein mislocalization of both Cyclin E and Gem, similar to knockdown of *lin19* (Figure 5G,H,J,K,M,N,P,Q). These cells were also co-stained with DAPI to visualize the nuclear region (Figure S1A,B,D,E,G,H,J,K). To validate the anti-Cyclin E antibody, we stained *Cyclin E RNAi* ^*JF02473*^ knockdown hemolymph samples. Cyclin E staining was decreased in enlarged cells from the knockdown genetic background, suggesting that the RNAi, as well as the antibody, function properly (Figure 5S and Figure S1 M). Interestingly, anti-Gem and DAPI staining in the knockdown of Cyclin E showed a similar localization to that of the SCF complex knockdown (Figure 5T and Figure S1 N). 

Previous research has described the requirement for Gem in the nucleus to inhibit Dup, a mechanism necessary for S-phase exit [26]. Together, these data suggest that Cyclin E plays a role in phosphorylating the substrate, possibly Dup, prior to SCF ubiquitination, due to the lack of the inhibitory protein Gem in the nucleus. To determine the association between this enlarged cell phenotype and Dup localization, *lin19* was knocked-down and hemolymph samples were stained with anti-Dup antibody. Wild-type hemocytes showed cytoplasmic staining of Dup, when compared to nuclear DAPI staining (Figure 5C,C’), while hemocytes from *lin19* RNAi knockdown had increased nuclear Dup localization as shown by colocalization with DAPI (Figure 5F,F’). Similar to Cyclin E and Gem staining, knockdown of the remainder of the SCF complex elicited a similar staining pattern as *lin19* knockdown, where there was increased Dup staining in the nucleus as visualized by increased antibody staining in the nuclear region (Figure 5I,L,O,R and Figure S1 C,F,I,L). Interestingly, in the knockdown of Cyclin E, there was also increased nuclear staining of Dup (Figure 5U and Figure S1 O). This suggests that Cyclin E may phosphorylate Dup in the nucleus, leading to Dup degradation. Together, all these data suggest that the SCF^Skp2^ complex ubiquitinates Dup, marking it for proteosomal degradation, and thus allowing for normal cell cycle progression of plasmatocytes.

### Overexpression of Gem in the SCF Knockdown Background Partially Rescues the Aberrant Plasmatocyte Phenotypes Caused by SCF Complex Knockdown

**Figure 6:**
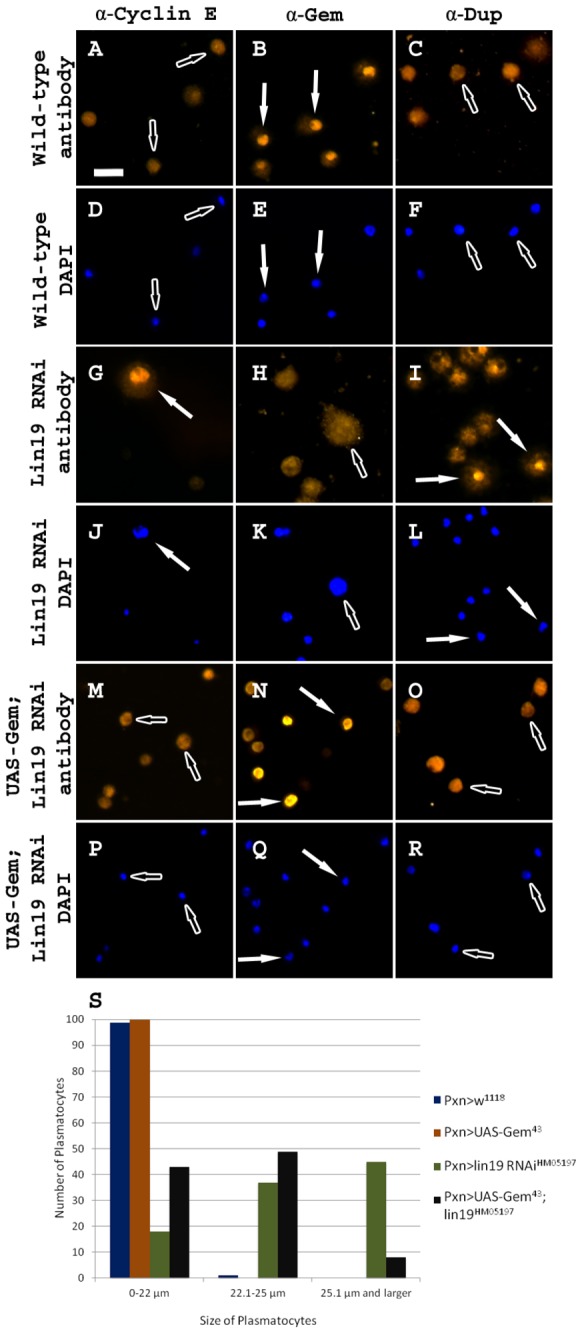
Overexpression of Gem elicits a partial rescue of SCF knockdown plasmatocyte phenotypes. (A-C) *pxnGal4>w* ^*1118*^ control hemolymph samples stained with (A) α-Cyclin E, (B) α-Gem, and (C) α-Dup and (D-F) DAPI. (G-I) *pxnGal4>UAS-lin19* *RNAi* ^*HM05197*^ hemocytes stained with (G) α-Cyclin E, (H) α-Gem, and (I) α-Dup and (J-L) DAPI. (M-O) *pxnGal4> UAS-Gem* ^*43*^ *; UAS-lin19* *RNAi* ^*HM05197*^ hemocytes stained with (M) α-Cyclin E, (N) α-Gem, and (O) α-Dup and (P-R) DAPI. (S) Graph indicating the number of cells that fall into specific size categories for *pxnGal4>w* ^*1118*^, *pxnGal4>UAS-Gem* ^43^, *pxnGal4>UAS-lin19* *RNAi* ^*HM05197*^, and *pxnGal4>UAS-Gem* ^*43*^ *; UAS-lin19* *RNAi* ^*HM05197*^ genotypes. As indicated by the graph, overexpression of Gem in the SCF knockdown background causes a great reduction in the number of giant cells when compared to knockdown of the SCF complex only. Nuclear stained cells indicated by white filled arrows, cytoplasmic stained cells indicated by outlined arrows. Scale bar = 20 μm.

To further investigate the role of Dup in the cell cycle of plasmatocytes, we overexpressed Gem, the inhibitor of Dup. It was hypothesized that by overexpressing Gem, we would be able to rescue the aberrant cell cycle phenotypes seen in the knockdown of SCF complex members. This rescue would occur by the inhibition of Dup by Gem, instead of the prominent mechanism of ubiquitination of Dup by the SCF complex. Wild-type hemolymph samples had cytoplasmic Cyclin E staining (Figure 6A,D), while Gem localized to the nucleus when visualized along with DAPI staining (Figure 6B,E). Consistent with previous results, Dup protein was also located throughout the cytoplasm of wild-type plasmatocytes (Figure 6C,F). As previously described, the subcellular localization of these proteins was altered when members of the SCF complex were abrogated, as seen in the *lin19 RNAi* functional knockdown. *lin19* knockdowns had nuclear Cyclin E (Figure 6G,J), along with Dup shown by co-localization with DAPI 

(Figure 6I,L), while Gem was removed from the nucleus (Figure 6H,K). It was also clear that a subset of the plasmatocytes in these hemolymph samples became vastly enlarged, likely by the mechanism of re-replication (Figure 6G-I). 

We then performed the overexpression of Gem in the *lin19 RNAi* background to assess if this would rescue the enlarged cell phenotype, as well as the aberrant cell cycle protein localization. Gem overexpression in *lin19* knockdown larvae caused Cyclin E protein to remain cytoplasmic, thus mimicking the phenotype of wild-type hemocytes (Figure 6M,P). Furthermore, overexpression of *Gem* caused the Gem protein to increase in the nuclear region, similar to the wild-type. However different from controls, the antibody staining suggested that there is an over-abundance of the Gem in the nucleus 

(Figure 6 N,Q). Finally, and most importantly, the majority of Dup protein was exported from the nucleus into the cytoplasm away from DAPI signal, again reminiscent of wild-type plasmatocytes 

(Figure 6O,R). We also observed a small number of cells which were decreased in size, but had a nuclear localization of Dup. It is possible that the cell size was rescued by the overexpression of Gem, however Dup is still localized to the nucleus, similar to the large cells in the SCF knockdown. 

Noticeably, hemolymph samples with overexpression of *Gem* using *UAS-Gem* ^43^ in the SCF knockdown background contained a decrease in giant cells (8/100 over 25.1 μm), with an increase in medium (43/100 under 22 μm) or slightly larger cells (49/100 were 22.1-25 μm) as assayed by anti-P1 plasmatocyte-specific antibody staining using our method of cell size counting (Figure 6S). All cells not stained by P1 were excluded. In comparison, *pxnGal4>UAS-lin19 RNAi* ^*HM05197*^ had 45/100 giant cells, with 37/100 slightly larger cells and only 18/100 medium cells. It was also noted that there was a statistically significant decrease in the average size of plasmatocytes in hemolymph samples from 26.25±5.14 to 22.65±2.07 (p<0.001). This is indicative that the size of the large cells caused by knockdown of the SCF complex, and lack of ubiquitination of Dup, is being partially rescued by the overexpression of Gem.

## Discussion

The generation of an *eaterGal4; UAS-GFP* strain allowed us to identify the functional importance of SCF complex members for the plasmatocyte blood cell lineage by a RNAi knockdown approach. Using this technique, we obtained hemolymph samples and were able to identify several genes belonging to the core SCF complex that, when knocked-down, caused a very distinctive giant cell phenotype. Importantly, as we were using *eater* as a driver to identify complex components, it was confirmed that these enlarged cells were plasmatocytes by anti-P1 plasmatocyte-specific antibody staining. This suggested to us, as proof-of-principle, that knockdown of gene function in mature plasmatocytes could elicit aberrant phenotypes dependent on the functional requirement of an essential gene/gene complex.

Previous research has shown that there are several *Drosophila* genes that may be involved in SCF complexes in order to determine specificity for a substrate. The F-box is thought to convey specificity of this complex by recruiting the substrate, however activation of the Cullin by neddylation factors also plays a role in ubiquitation of the substrate [28-30]. A comprehensive list of all known and predicted complex members has been published [31]. We used this list to identify the remaining members of the specific SCF complex that function in *Drosophila* hematopoiesis, as knockdown of only one of each of the core components caused enlarged plasmatocytes (Table S2). We have shown that *lin19, SkpA* and *Roc1a* likewise play a role in the hematopoietic niche, the PSC of the larval lymph gland. Knockdown of these genes caused a decrease in the number of PSC cells, as well as an increase in the size of these cells [21]. These data, along with the findings in this current study, suggest that the SCF complex has a significant role in multiple aspects of *Drosophila* larval hematopoiesis.

Using fluorescence microscopy, it was noted that the enlarged cells caused by the SCF knockdown had a significant excess of DNA in the nuclear region. To investigate the hypothesis that DNA re-replication was occurring in plasmatocytes with the SCF complex knockdown, anti-gamma- Tubulin staining of centrioles was performed. Previously, it was shown that knockdown of Gem elicits DNA re-replication, therefore we used it as a positive control [26]. It was evident through our experiments that the *lin19* knockdown had multiple centrioles in one giant plasmatocyte, similar to plasmatocytes from the *gem RNAi* samples. It was also clear that the DNA had replicated many times, without any cellular division as indicated by BrdU-positive, but phospho-Histone H3-negative enlarged cells. These data support the idea that plasmatocytes from SCF knockdown animals undergo DNA re-replication, thus the SCF complex is necessary for Dup degradation. Additionally, previous research had identified a number of proteins that when misexpressed or knocked-down cause an enlarged cell phenotype with excess DNA replication [26,27,32-34]. Several papers have shown that misregulation of Cyclin E can cause aberrant DNA synthesis [27,34]. Research has also suggested that knockdown of Gem can cause this excessive DNA phenotype [26]. In our experiments, antibody staining identified that the subcellular localization of both these proteins changed between control samples and the *lin19* knockdown. Importantly, Dup is necessary for DNA replication, but it must be degraded to prevent re-replication [32,33]. As the main role of Gem is to inhibit Dup, and Gem was no longer found in the nucleus in the knockdown, this is suggestive that Gem had complexed with Dup, removing it from the nucleus [26]. Conversely, Cyclin E was found in the nucleus. This is notable because Cyclin E is known to phosphorylate Dup marking it for ubiquitination, leading to its nuclear localization [34]. It is also known that SCF^Skp2^ degrades Cyclin E [35]. This is another explanation for the accumulation of Cyclin E in the nucleus of SCF knockdown hemolymph samples. These data suggest that Dup may be the target substrate for the SCF complex being studied, with a secondary target possibly being Cyclin E. Previous research in human cells has shown that SCF^Skp2^ regulates the degradation of Cdt1 (the homolog of *Drosophila* Dup) [32]. It has also been shown that the activated SCF^Skp2^ complex plays a role in murine hematopoiesis, by ubiquitinating proteins necessary for proper cell cycle, such as Cyclin E. There are still many questions to be answered about SCF regulation in blood cells, as some of these results are contradictory [36-38].

 In addition to these data, protein localization in the knockdown of Cyclin E showed that Gem had been removed from the nucleus, again consistent with the notion that it was titrated away from the nucleus by binding Dup. This is plausible because the SCF complex can recognize its substrates due to phosphorylation state [39,40]. Since Cyclin E was knocked-down, Dup was not properly phosphorylated, and it was not recognized as the substrate by the SCF complex, therefore never being ubiquitinated nor degraded. Furthermore, in the Cyclin E knockdown, Dup localized to the nucleus similar to its localization in the SCF knockdown. This would make it necessary for Gem to inhibit Dup, causing Gem to take on a non-nuclear localization, while Dup would have a nuclear localization, if Dup was in excess. Taken together, these lines of investigation support the hypothesis that Cyclin E is necessary to phosphorylate Dup, allowing the SCF complex to recognize and ubiquitinate it. Dup that remains in the nucleus after degradation must be bound by Gem for the cell cycle to progress properly [26,41,42]. DNA re-replication will occur if Dup remains in the nucleus [27,34]. It is highly suggestive that knockdown of Cyclin E or the SCF complex perturbs this mechanism, causing Dup accumulation in the nucleus, and the cells to re-initiate DNA replication. Furthermore, others have shown there must be a balance of Gem and Dup in the nucleus for proper progression through the cell cycle [43-45]. Our research shows that there is a lack of Gem and an accumulation of Dup in the nucleus, which leads to excessive DNA replication and additional centriole replication in five percent of the plasmatocyte population. 

Although re-replication is one mechanism to explain the SCF loss-of-function phenotype, a similar non-canonical process, known as endoreplication, could also account for the over-replicative system in these cells. Endoreplication is a cycle in which cells undergo S phases that are separated only by gap phases but not an intervening mitosis [46,47]. However, endoreplication is not known to occur in wild-type *Drosophila* plasmatocytes. Further, *Drosophila* plasmatocytes are most similar to mammalian macrophages, which also do not endoreplicate [48]. Since several of the proteins studied in this paper have been implicated in re-replication with phenotypes including enlarged cells, increased DNA content, and multiple centriole replication, we favor the hypothesis that re-replication is triggered in plasmatocyte development in the absence of SCF complex activity [25–27,32,34,45].

It is intriguing that only five percent of the cells display the re-replication phenotype, and we have several hypotheses on this point. One explanation is that the smaller cells have arrested. There are many intrinsic mechanisms to ensure proper cell cycle progression preventing re-replication and ultimately cancer. It is possible these enlarged cells have escaped these mechanisms, causing the cell to replicate their DNA many times without going through mitosis, while the smaller cells arrest, to prevent this phenotype. It is also possible that only five percent of these cells are going through cell division during the time the RNAi is functionally knocking down the gene. Previous research has suggested that during mid-to-late third instar larval stages, only one to two percent of cells are going through mitosis at a given time [5]. *eaterGal4* is activated during second instar, however there is likely a latent period between activation of Gal4 and protein knockdown by the RNAi. This is consistent with only five percent of cells having an active cell cycle, and becoming enlarged through re-replication. A final possibility is that there are partially redundant mechanisms for the regulation of Dup. As previously described, the SCF complex has been shown to be involved in the ubiquitination and subsequent degradation of Dup, and Gem will inhibit the remainder of the Dup that may be in the nucleus [26,27,34]. There may be additional mechanisms which ubiquitinate or inhibit Dup, therefore avoiding re-replication. The smaller cells may have activated one of these mechanisms to aid the cell in proper cell cycle, ultimately avoiding cancer. The regulation of Dup is of vast importance, and there are several possibilities of alternate mechanisms to prevent the re-replication phenotype elicited by cells which have excess Dup in the nucleus.

To further implicate the necessity of Dup regulation in the proper cell cycle of plasmatocytes, a rescue experiment was performed by overexpressing the Dup inhibitor, Gem. By overexpressing this inhibitory protein, it was hypothesized that the nuclear localization of Gem would increase, the protein would bind Dup, and therefore decrease the re-replication that is observed in SCF complex knockdown. Performing immunohistochemistry experiments identified that there was an increase in nuclear Gem and a decrease in Dup. Additional experimental evidence supports this hypothesis as there is a decrease in size of plasmatocytes with genotype *pxnGal4>UAS-Gem* ^*43*^ *>UAS-lin19 RNAi* ^*HM05197*^ compared with *pxnGal4>UAS-lin19 RNAi* ^*HM05197*^. There is a drastic decrease in the number of giant cells, which are larger than 25.1 μm, in *pxn>UAS-Gem* ^*43*^ *>UAS-lin19 RNAi* ^*HM05197*^ (8/100) plasmatocytes compared with SCF knockdown hemocytes (45/100). It was also noted that there was a significant decrease in the average size of plasmatocytes in hemolymph samples from *Gem* overexpression in the SCF knockdown background (p<0.001). These lines of evidence are all suggestive that knockdown of the SCF complex increased nuclear Dup leading to re-replication. By over-expressing its inhibitor, Gem, it is possible to partially rescue this enlarged cell phenotype generated by excess nuclear Dup. These data suggest the regulation of Dup is important in the proper cell cycle progression of plasmatocytes. Furthermore, these data support the hypothesis that the SCF^Skp2^ complex is responsible for the ubiquitination of Dup, allowing plasmatocytes to proliferate properly. Although this study provides substantial genetic evidence that the SCF^Skp2^ complex is necessary to ubiquitinate Dup allowing for proper hematopoietic cell cycle progression, future studies using biochemical techniques to show physical interactions are needed to support the model proposed here.

Furthermore, there are two ubiquitin ligase complexes known to be involved in the ultimate degradation of Dup: The SCF^Skp2^ complex, described in this manuscript, and the Cul4-DDB1-CDT2-PCNA (Cul4^CDT2^) complex [32,49,50]. To vastly decrease the possibility that the Cul4^CDT2^ complex was responsible for the enlarged cell phenotype, both DDB1 and PCNA were knocked-down via RNAi and Cul4 mutants were also analyzed. Although DDB1 functional knockdown elicited a small number of enlarged cells, these cells had a different morphology than the SCF^Skp2^ knockdown (data not shown). Additionally, none of the other analyses elicited any enlarged cells as observed when the SCF^Skp2^ complex was knocked-down. This further implicates the necessity of the SCF^Skp2^ complex in the proper plasmatocyte cell cycle.

**Figure 7:**
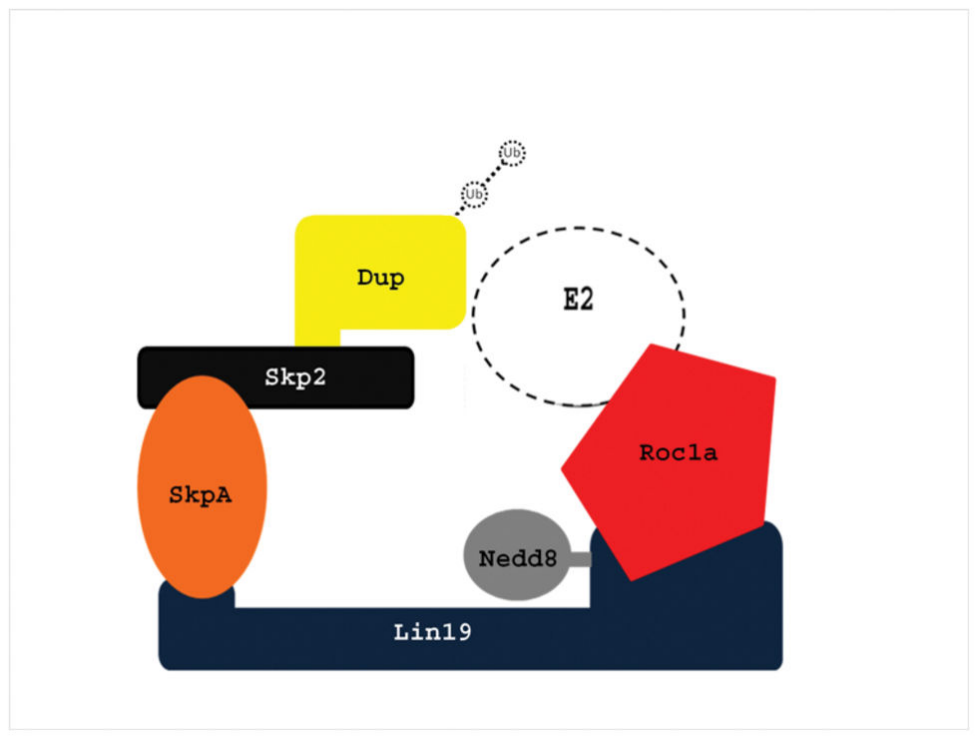
Model of the SCF complex involved in *Drosophila* plasmatocyte development. The SCF complex that functions in hematopoiesis consists of Lin19, SkpA, Skp2, Roc1a and Nedd8. This complex functions to ubiquitinate its substrate, Dup, which is necessary for the proper cell cycle progression of plasmatocytes.

To summarize, in this manuscript we identify the SCF ubiquitin ligase complex as a novel regulator of plasmatocytes. Genetic evidence is presented that suggests that Dup is the main target for the SCF^Skp2^ complex (Figure 7). We propose that the SCF^Skp2^ complex plays an integral role in *Drosophila* hematopoiesis by ubiquitinating Dup, which is necessary for proper cell cycle progression. Knockdown of the SCF complex causes an accumulation of Dup in the nucleus, inducing the cell to undergo multiple rounds of replication without an intervening mitosis or cytokinesis. This causes some plasmatocytes to become vastly enlarged, with multiple centrioles and excessive DNA content. Taken together, our findings provide evidence that the SCF complex is necessary for proper cell cycle progression during plasmatocyte development in *Drosophila*. As the SCF complex is conserved from *Drosophila* to humans, these findings implicate the importance of the roles of ubiquitin ligase complexes in the cell cycle and their potential malfunctions in blood cell cancers.

## Materials and Methods

### *Drosophila* Strains and Culture

Fly lines were cultured at room temperature or 18°C and crosses were maintained at 25°C on standard fly food. RNAi and mutant stocks, listed in tables S1 and S2, were obtained from the Bloomington Stock Center and the Vienna *Drosophila* RNAi Stock Center. Also used were *w* ^1118^ from the Bloomington Stock Center, and *PxnGal4* (M. Galko; [51]), 2-1 *UAS-Gem* (B. Calvi; [52]) and *UAS-Gem* ^43^ (H. Richardson; [53]). *eaterGal4>UAS-GFP* flies were generated and used to analyze RNAi stocks [15,20].

### Immunostaining of *Drosophila* Circulating Hemocytes

Larval hemocyte samples were obtained as described previously [15]. The following primary antibodies were used: rat anti-Gem (1:1000; gift from B. Calvi; [25]), guinea pig anti-Dup (1:5000; gift from T. Orr-Weaver; [54]), mouse anti-Cyclin E 8B10 (1:40; gift from H. Richardson; [55]), mouse anti-P1 (1:200; gift from I. Ando; [56]), mouse anti-acetylated-Tubulin (1:10,000; Sigma item # T-6793), rabbit anti-gamma- Tubulin (1:50,000; Sigma item # T-5192). Secondary antibodies used were mouse, guinea pig, rat and rabbit Alexafluor 488, 555 or 610 (1:500; Invitrogen). Samples were also incubated with DAPI (1:500) and analyzed with a Zeiss Axioplan fluorescence microscope or Nikon AR-1 laser-scanning confocal microscope. Images were processed with ImageJ, Adobe photoshop or 3D blind deconvolution using AutoQuant X^2^ software (Media Cybernetics, Rockville, MD). 

### BrdU Staining of *Drosophila* Plasmatocytes

BrdU was diluted in standard fly food at 1 mg/ml (Sigma Item #B5002). Flies were maintained on this media for 3 days, when the adults were removed, and vials placed at 25°C. Hemocytes were obtained as previously described and stained with DAPI (1:500) and anti-BrdU (1:500; Sigma Item #B2531) [15].

### Counting of Enlarged Plasmatocytes

Hemocyte samples were obtained as previously described [15]. Samples were stained using anti-P1 (1:200; gift from I. Ando [56]) followed by Alexafluor anti-mouse 555 (1:500; Invitrogen). Images were obtained from each genotype with a Zeiss Axioplan fluorescence microscope and the diameter of cells larger than 18 μm from each field of view were measured and recorded.

## Supporting Information

Figure S1 **DAPI staining of enlarged cells induced by SCF component RNAi knockdown.** Hemocytes from Figure 4 were DAPI stained to identify the nuclear region. (A-L) Visualization of the nucleus using DAPI staining of the remaining components of the SCF complex. The results recapitulate the data observed in *pxnGal4>UAS-lin19* RNAi knockdown showing enlarged cells have increased DNA content. (M-O) DAPI staining of *pxnGal4>UAS-Cyclin* *E* *RNAi* ^*JF02473*^ indicating knockdown of Cyclin E, cytoplasmic staining of anti-Gem and nuclear staining of anti-Dup.
(TIF)

Table S1 **RNAi stocks used to analyze knockdown of SCF complex function.** A list of RNAi stocks used to determine and verify knockdown of the SCF complex that caused the enlarged plasmatocyte phenotype.
(XLSX)

Table S2 **List of stocks used to determine members of the SCF complex functioning in *Drosophila* hematopoiesis.** The majority of possible SCF complex members were knocked-down via RNAi, with the remaining possible members screened by mutant analysis. The analysis identified knockdown of one member of each of the core components induced the enlarged cell phenotype, shown in bold.
(XLSX)
