# Supplementary figures and images for: Knockdown of SCFSkp2 Function Causes Double-Parked Accumulation in the Nucleus and DNA Re-Replication in Drosophila Plasmatocytes
Source: PLoS One. 2013 Oct 24;8(10):e79019. doi: 10.1371/journal.pone.0079019 (PMC3812016; doi:10.1371/journal.pone.0079019)

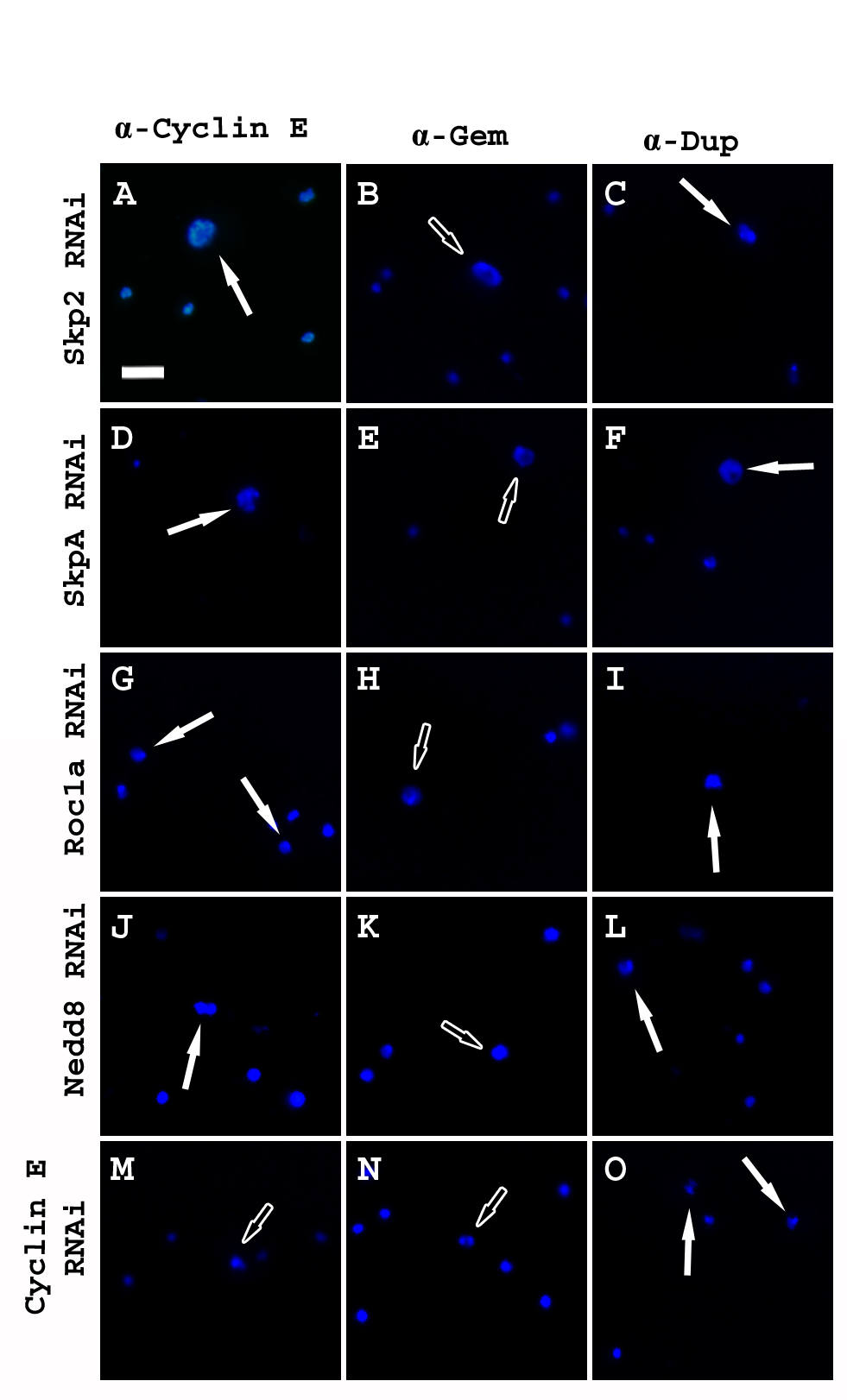

Supplement: Figure S1 — DAPI staining of enlarged cells induced by SCF component RNAi knockdown. Hemocytes from Figure 4 were DAPI stained to identify the nuclear region. (A-L) Visualization of the nucleus using DAPI staining of the remaining components of the SCF complex. The results recapitulate the data observed in pxnGal4>UAS-lin19 RNAi knockdown showing enlarged cells have increased DNA content. (M-O) DAPI staining of pxnGal4>UAS-Cyclin E RNAi JF02473 indicating knockdown of Cyclin E, cytoplasmic staining of anti-Gem and nuclear staining of anti-Dup. (TIF) [file pone.0079019.s001.tif]
